# Can high pain intensity and catastrophizing interfere with the cognitive performance of women with chronic pain related TMD? A cross-sectional study

**DOI:** 10.1590/1678-7757-2022-0384

**Published:** 2023-03-27

**Authors:** Melissa de Oliveira MELCHIOR, Luiza Guilherme ANTUNES, César BATAGLION, Laís Valencise MAGRI

**Affiliations:** 1 Universidade de São Paulo Faculdade de Odontologia de Ribeirão Preto Ribeirão Preto SP Brasil Universidade de São Paulo, Faculdade de Odontologia de Ribeirão Preto, Ribeirão Preto, SP, Brasil.; 2 Universidade de Ribeirão Preto Ribeirão Preto SP Brasil Universidade de Ribeirão Preto, UNAERP, Ribeirão Preto, SP, Brasil.

**Keywords:** Facial pain, Pain intensity, Cognitive performance, Catastrophizing, Hypervigilance, Central Nervous System Sensitization

## Abstract

Interventions based on pain education and self-management are dependent on factors such as attention, memory, concentration, and executive function.

**Objective:**

To explore the relationship between cognitive performance and the variables pain intensity, central sensitization, catastrophizing, and hypervigilance in women diagnosed with chronic pain-related TMD.

**Methodology:**

This is a cross-sectional study. A total of 33 women (mean age: 38±4.6 years; range: 18 to 66 years) with chronic pain-related TMD (myalgia and/or arthralgia) diagnosed according to the Diagnostic Criteria for Temporomandibular Disorders (DC/TMD). Specific questionnaires were used to evaluate cognitive performance, overall pain intensity, central sensitization, hypervigilance, and pain catastrophizing. The data were analyzed using Pearson’s correlation coefficient and backward stepwise multiple linear regression (statistical significance at 5% alpha).

**Results:**

Approximately 53% of the study sample showed decreased cognitive performance. High central sensitization, hypervigilance, and pain catastrophizing were observed. A significant negative correlation was observed between cognitive performance and hypervigilance (p=.003, r=−.49), cognitive performance and catastrophizing (p<.001, r=−.58), and cognitive performance and pain intensity (p<.001, r=−.58). Regarding the partial regression coefficients, only catastrophizing and pain intensity showed statistical significance (t=−2.12, p=.043; t=−2.64, p=.014, respectively), indicating a significant role in explaining cognitive performance at the sample.

**Conclusion:**

High pain intensity and the presence of catastrophic thoughts regarding pain can predict impaired cognitive performance in women with chronic pain-related TMD. Management strategies addressing psychosocial dimensions such as reducing catastrophizing and ensuring complete understanding of the condition are important.

## Introduction

The high prevalence of chronic pain and the therapeutic difficulties associated with its management makes it a serious public health problem^1 , 2^ with considerable social, familiar, emotional, and cognitive impacts. Therefore, the management of chronic pain must go beyond the individual experience to include a wider approach that takes nociception and other bio-psychosocial aspects into consideration.^3^ Both in Brazil and worldwide, chronic pain is more commonly observed in women, and its prevalence rates range from 29% to 73%,^4^ creating a need for health services that focus on the development of specific management strategies aimed at prevention and intervention.

The current understanding of temporomandibular disorders (TMD) is based on previous evidence that identifies it as a multi-systemic alteration resulting in generalized chronic pain.^5 , 6^ Although current interventions based on pain education and self-management are dependent on factors such as attention, memory, concentration, and executive function, all of which play a crucial role in the long-term maintenance of well-being, the literature lacks evidence on the cognitive performance of patients diagnosed with this condition.

Patients with chronic pain typically exhibit impaired outcomes in tests examining cognitive performance and self-perception of their condition in social situations and daily activities.^7 - 9^ Previous observational clinical studies found that most patients with chronic pain report loss of memory and concentration, particularly during severe episodes.^8 , 10 , 11^

Other studies showed that activities related to the executive function may also be mild to moderately impaired in these patients, leading to higher levels of distraction and reduced cognitive abilities.^1 , 12 , 13^ The literature also shows the significant role of memory in the relationship between chronic pain and cognitive performance, particularly in older patients, although the specific type of memory (working memory, short and/or long-term memory, or autobiographical memory) involved is still unclear.^1 , 14^

The experience of pain also includes various emotional and behavioral aspects, such as catastrophizing and hypervigilance, which can influence pain modulation and hinder effective management.^15 , 16^ Therefore, this study aimed to explore the relationship between the variables pain intensity, central sensitization, catastrophizing, and hypervigilance with cognitive performance in women diagnosed with chronic pain-related TMD (myalgia and/or arthralgia) to elucidate the influence of those variables on the cognitive performance and to contribute to the development of appropriate strategies that consider these parameters. The hypothesis is that cognitive performance is influenced by these predictor variables.

## Methodology

### *Study design:* Analytical, observational, cross-sectional study design used.

#### Approval from the Research Ethics Committee

Ethical approval was obtained from the research ethics committee of the School of Dentistry, Ribeirão Preto, University of São Paulo (FORP/USP) (CAAE 03383218.7.0000.5419), and all study participants were required to provide informed consent, according to the ethical standards of the Regulatory Norms for Research in Humans, Resolution 466/2012, CONEP, Brazil.

#### Sample

The sample was established for convenience, based on the number of patients received at the service (a total of 52) in 2019 who met the established inclusion criteria. The study sample included 33 women (mean age: 38±14.6 years; range: 18 to 66 years), recruited at the TMD graduate clinic of FORP/USP during 2019, with various diagnoses of chronic pain-related TMD (myalgia, headache attributed to TMD, and/or arthralgia) for six months or more, according to the Diagnostic Criteria for Temporomandibular Disorders (DC/TMD)^17 , 18^ translated and validated for the Brazilian-Portuguese population.^19^ Patients with a history of tumors, psychiatric, or neurological diseases that compromised cognitive performance, and/or major surgeries in the stomatognathic system were excluded as these could all be potential sources of chronic pain and, therefore, possible confounders. Demographic data including age, level of education, duration of pain (in months), presence of comorbidities, and chronic use of medications were recorded.

#### Evaluation instruments

*Measurement of cognitive performance: MoCA test*

The MoCA (Montreal Cognitive Assessment) screening test for mild cognitive deficit contains eight key domains: visuospatial/executive, naming, memory, attention, language, abstraction, delayed recall, and orientation.^20 , 21^ Its score can be used to predict the cognitive competencies of the individual, with a total score <26 indicating cognitive impairment. In this study, the test was conducted by a calibrated evaluator, with the estimated time of execution being 10 minutes.

*Pain Assessment–independent variables*

Pain perception, catastrophizing, hypervigilance, and central sensitization were assessed using specific validated questionnaires. The perception of their overall pain intensity in the previous week was assessed using a visual analog scale, in which the participant was asked to mark a number ranging from zero (absence of pain) to 10 (worst possible pain) to describe their pain experience.

The 13 items of the Brazilian Portuguese version of the Pain Catastrophizing Scale [(BP)-PCS],^15^ a self-administered questionnaire used. The patients were asked to mark scores [range: 0 (almost never) to 5 (almost always)] to describe their thoughts or feelings, with a higher final score indicating a greater level of pain catastrophizing (Cutoff score of 30 can be used to generally indicate the presence of this characteristic and was used in descriptive analysis - Table 3 ).

**Table 3:** Sample number (n), mean (standard deviation) of MoCA score (cognitive performance), VAS score (pain intensity), total CSI score (central sensitization), total PVAQ score (hypervigilance), and total PCS score (catastrophizing) by clusters of mild (VAS 1–3), moderate (VAS 4–6), or severe (VAS 7–10) pain intensity. Central sensitization indicated by CSI>40; hypervigilance indicated by PVAQ>40; pain catastrophizing indicated by PCS>30)

	n	VAS	CSI	PVAQ	PCS	MoCA
Total Sample	33	6 (±2)	41.32 (±16)	48 (±12)	24 (±13)	24 (±3)
VAS (1–3)	2	3 (±0)	50.50 (±6)	34 (±15)	18 (±3)	26 (±3)
VAS (4–6)	15	5 (±1)	42.4 (±12)	45 (±10)	18 (±10)	27 (±3)
VAS (7–10)	16	8 (±1)	39 (±20)	53 (±12)	31 (±13)	22 (±3)
CSI ≤ 40	18	6.5 (±2)	29 (±7)	45 (±11)	24 (±13)	25 (±3)
CSI > 40	15	6 (±2)	56 (±10)	52 (±13)	24 (±13)	24 (±4)
PVAQ ≤ 40	7	5 (±2)	34 (±8)	31 (±7)	18 (±14)	27 (±3)
PVAQ > 40	26	7 (±2)	44 (±17)	53 (±9)	26 (±13)	24 (±3)
PCS ≤ 30	20	6 (±2)	40 (±14)	45 (±10)	15 (±7)	26 (±3)
PCS > 30	13	8 (±2)	45 (±20)	55 (±14)	38 (±5)	22 (±3)

VAS: Visual Analogue Scale; CSI: Central Sensitization Inventory; PVAQ: Pain Vigilance and Awareness Questionnaire; PCS Pain Catastrophizing Scale; MoCA Montreal Cognitive Assessment.

Pain hypervigilance was evaluated using the Pain Vigilance and Awareness Questionnaire (PVAQ - Brazilian Portuguese),^16^ which consists of 16 items that are scored using a Likert scale [range: 0 (never) to 5 (always)]. The items represent the degree to which each description of pain behavior was experienced by the patient in the two weeks before the study. Although pain hypervigilance can be classified using the 25^th^, 50^th^, and 75^th^ percentiles of the total scores for each of the PVAQ factors, this method was not used in this study because the aim was to examine correlations and evaluate the influence of different variables on cognitive levels. Cutoff score of 40 can be used to generally indicate the presence of hypervigilance and was used for descriptive analysis ( Table 3 ). As chronic pain affects the nociceptive circuit of the central nervous system, the participants’ central sensitization was assessed using the CSI questionnaires (Central Sensitization Inventory).^21^ The first part of these questionnaires (Part A) assessed 25 signs and symptoms related to central sensitization, with specific statements being assigned scores on a Likert scale [range: 0 (never) to 4 (always)]. The total score can range from 0 to 100, with 40 points or more being an indicative of painful central sensitization. The second part of the questionnaires (Part B) was composed of questions on the presence of confirmed diagnoses associated with central pain sensitization.

## Statistical analyses

The post-study power test was used to calculate the scope power of the study using the website clincalc.com. It was based on the cutoff value for the normality of the cognitive performance test, whose value is 26, on the average value found in the studied sample (24), with alpha value set at 0.05. The achieved value of post-hoc power for the sample was 99.6%. This value should be considered with caution, since we found no studies on the application of this test in people with TMD; therefore, the mean of this variable for this population is unknown. Considering this, the following statistical methodology was applied:

First, the sample was analyzed according to the DC/TMD diagnosis of pain-related TMD conditions (myalgia and/or arthralgia) to verify if the variables analyzed in this study differed between the groups of myalgia and/or arthralgia, or if these diagnostic groups could be analyzed together. Headache attributed to TMD was found associated with other pain-related TMD in the studied sample and was not considered as a cluster in this study. After confirmation of normality for all study variables using the Shapiro–Wilk test (p>.05), Pearson’s correlation and backward stepwise multiple linear regression tests were used to analyze the data. An alpha level of 5% was considered statistically significant, and all analyses were conducted using the Bioestat 5.3 program. The Pearson’s correlation coefficient was categorized into weak correlation (0 to 0.4), moderate correlation (0.5 to 0.7), and strong correlation (0.8 to 1). The null hypothesis (H0) was that the cognitive performance of women with chronic pain-related TMD was not influenced by pain intensity, hypervigilance, catastrophizing, and/or central sensitization [β1=0, β2=0, β3=0, β4=0]. The alternative hypothesis (H1) was that the cognitive performance of women with chronic pain-related TMD was influenced by pain intensity, hypervigilance, catastrophizing, and/or central sensitization [at least one β1≠0].

## Results

**Figure 1:**
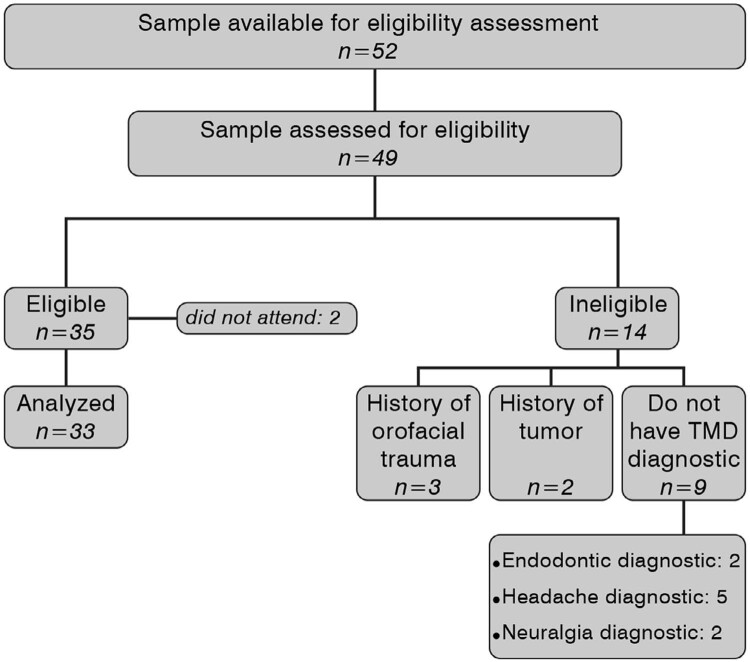
Flowchart of sample selection steps, following the inclusion and exclusion criteria; eligible and non-eligible volunteers and losses incurred for various reasons

A total of 52 people were directed to the service of TMD and 49 of them, all women, were eligible for this study. The number of participants reached was 33 women. The Flowchart below ( Figure 1 ) shows the process of defining the final sample achieved.

In accordance with the DC/TMD,^17 , 18^ temporomandibular joint disorders were classified into 12 categories, as follows: local myalgia (7), myofascial pain (7), referred myofascial pain (15), arthralgia (26), TMD attributed headache (14), disc displacement with reduction (11), disc displacement with reduction with intermittent locking (1), degenerative disease (5), and subluxation (1). The duration of pain ranged from 6 to 420 months (mean duration: 80.24±85 months). After the TMD diagnosis, the sample was distributed in pain-related TMD clusters, as follows: four subjects with arthralgia, seven subjects with myalgia, and 22 subjects with arthralgia and myalgia associated. Table 1 shows descriptive data (mean, standard deviation) by cluster and an analysis of variation (ANOVA 1 criterion) between them for each variable studied. The results showed no significant differences between clusters (p>.05).

**Table 1:** Mean (standard deviation) of the studied variables according to the subgroups of pain-related TMD diagnoses. Analysis of variance (ONE-WAY ANOVA).

	Artralgia	Myalgia	A + M	ANOVA	
				F	p-value
MOCA^†^	26.75 (±3.40)	24.71 (±4.61)	24.41 (±3.11)	0.76	0.52
PVAQ^‡^	49.5 (±3.42)	46.57 (±15.87)	48.27 (±12.72)	0.07	0.93
PCS^§^	17.5 (±7.85)	19.29 (±13.47)	26.82 (±13.13)	1.53	0.23
CSI^¶^	29.25 (±12.42)	46.00 (±19.85)	42.32 (±15.31)	1.46	0.25
VAS^π^	5.75 (±2.06)	6.43 (±1.27)	6.41 (±2.11)	1.46	0.82

Arthralgia (A) and Myalgia (M): Diagnosed and classified according to the Diagnostic Criteria for the Most Common Temporomandibular Disorders: Symptom Questionnaire and Clinical Examination Items DC/TMD (Version 10/23/2015)*. Significance level: p<.05. MoCA: Montreal Cognitive Assessment; PVAQ: Pain Vigilance and Awareness Questionnaire; PCS: Pain Catastrophizing Scale; CSI: Central Sensitization Inventory; VAS: Visual Analogue Scale

Table 2 shows the level of education of the sample, as the number of school years is considered for the score on the cognitive performance test. People with less than 12 school years add one point to the final score of the test. The chronic medications used by the study sample included analgesics/anti-inflammatory drugs (n=13), central-action analgesics (n=5), antidepressants (n=8), benzodiazepines (n=4), and anticonvulsants (n=4).

**Table 2:** Level of education of the studied sample, sample number (n), Number of school years completed (No. School Years)

Level of education	n	No. School Years
Incomplete elementary school	8	<12
Complete elementary school	2	<12
Incomplete high school	2	<12
Complete high school	11	12
Incomplete higher education	6	>12
Complete higher education	4	>12

Note: own elaboration.

Table 3 shows the descriptive characteristics of the studied sample. Patients with perceptions of severe pain (VAS 7–10) exhibited higher levels of central sensitization (CSI), pain catastrophizing (PCS), and hypervigilance (PVAQ), and lower levels of cognitive performance (MoCA). Participants with CSI>40 had moderate VAS, marked hypervigilance, and poor cognitive performance (<26). Among the participants with high hypervigilance (PVAQ>40), the average for VAS showed severe pain perception, presence of central sensitization, and low cognitive performance (average <26). Cluster of pain catastrophizing scores above 30 showed severe perception of pain intensity (VAS), evidence of central sensitization (CSI>40), hypervigilance, and poor cognitive performance. Approximately 53% (n=18) of the study sample exhibited total MoCA scores lower than 26, indicating a deficit in cognitive performance.

**Figure 2:**
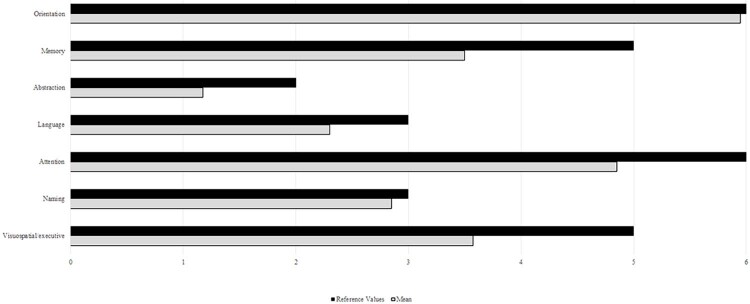
Reference values and mean values for the cognitive domains of the MoCA test

Regarding the MoCA domains, attention, memory, and visuospatial/cognitive function obtained the lowest reference values ( Figure 2 ). Language and naming were also slightly reduced.

### Pearson’s correlation and multiple linear regression analyses

A significant positive correlation was observed between hypervigilance and catastrophizing (p=.022, r=-.39), hypervigilance and pain intensity (p=.017, r=.41), hypervigilance and central sensitization (p=.004, r=.48), and catastrophizing and pain intensity (p=.013, r=.43). Central sensitization showed no significant correlation with cognitive performance and pain intensity (p=.104, p=.52, respectively).

A significant negative correlation was observed between cognitive performance and hypervigilance (p=.003, r=−.49), cognitive performance and catastrophizing (p<0.001, r=−.58), and cognitive performance and pain intensity (p<.001, r=−.56). The “r” values indicated weak to moderate correlations between all variables, and the majority were statistically significant (p<.05) ( Table 4 ).

**Table 4:** Pearson’s correlation analysis between ǂpredictors variables and response variables (cognitive performance)

ǂ Predictors	r	R2	p	IC−95%
Hypervigilance	-0.49	0.24	0.003	- 0.72 to -0.18
Catastrophizing	-0.58	0.34	0.0004	- 0.77 to -0.30
Central sensitization	-0.29	0.08	0.104	- 0.57 to 0.06
Pain intensity	-0.56	0.31	0.0007	- 0.76 to - 0.26

Note: Own elaboration.

Multiple linear regression analysis examining the influence of these sensory and behavioral variables on cognitive performance yielded a significant F value (regression) (p<.001); thus rejecting the null hypothesis and accepting the alternative hypothesis, which established that at least one of the independent variables influenced the cognitive performance of women with chronic pain-related TMD. Regarding the partial regression coefficients, only pain catastrophizing and pain intensity showed statistically significant results (t=−2.12, p=.043; t=−2.64, p=.14, respectively), indicating a significant role in explaining cognitive performance in women with chronic pain-related TMD.

A significant correlation between the predictor and response variables must be met to conduct backward stepwise multiple linear regression analysis. Therefore, central sensitization (CSI score) was removed from the model as it showed no statistically significant correlation with the response variable, cognitive performance (p=.104). Additionally, the partial regression coefficients for hypervigilance (b=−.021) and central sensitization (b=−.052) were not statistically significant (t=−.44, p=.66; t=−1.57, p=.13 respectively), indicating insufficient evidence to conclude that these variables were related to cognitive performance in the study sample. The model was then adjusted to include pain catastrophizing and pain intensity only, and Table 5 shows the results of the multiple linear regression models. Pain catastrophizing (PCS score) and pain intensity accounted for approximately 33.61% and 11.77% of the determination of the dependent variable, respectively. Together, they accounted for 45.38% of the cognitive performance of the sample studied (p<.001), suggesting that other factors were unlikely to influence the response variable.

**Table 5:** Backward stepwise multiple linear regression analysis for the dependent variable, cognitive performance [measured using the Montreal Cognitive Assessment (MoCA) questionnaires; n=33]

Model	Predictor	p	R2	Intercept	Beta	p-value
**1**						
	Hypervigilance (PVAQ)	0.0004*	0.4764	34.548	-0.0209	0.66
	Catastrophizing (PCS)				-0.0821	0.043*
	Central sensitization (CSI)				-0.0522	0.13
	Pain intensity (VAS)				-0.7542	0.014*
**2**						
	Catastrophizing (PCS)	0.0003*	0.4174	31.79	-0.1116	0.009*
	Pain intensity (VAS)				-0.6862	0.016*

*p<.05; p: significance of the multiple regression model tested; p-value: significance of partial regression coefficients; R2: coefficient of determination of the multiple regression model tested; Beta: partial regression coefficient; Intercept (or Constant): value of the response variable when predictor variables are equal to zero.

Finally, the linear model that considered catastrophizing (PCS) and pain intensity (VAS) for the prediction of Y can be represented by the following equation: Yˆ (MoCA) = 31.79 - 0.112 PCS - 0.69 VAS.

Based on these findings, cognitive performance (Y) should decrease by −0.112 for each unit of increase in catastrophizing score, and by −0.69 for each unit of increase in pain intensity score.

## Discussion

Our study showed significant correlation between pain intensity, catastrophizing, hypervigilance, and central sensitization, suggesting clinical relevance of these factors in women diagnosed with chronic pain-related TMD. The behavioral, cognitive, and emotional aspects of chronic pain have been recognized as crucial in the maintenance and aggravation of painful conditions,^5 , 6 , 22 , 23^ and the weak to moderate strength of correlation observed in this study agreed with previous literature when recognizes the multidimensionality of chronic pain and the varied contributions of several factors. We found a negative correlation between cognitive performance and the predictor variables, suggesting a possible influence of one or more factors on various aspects of cognitive performance. Backward stepwise multiple linear regression analysis was used to specifically elucidate dependency relationships, and the results showed that only pain catastrophizing (p=0.043) and pain intensity (p=0.029) contributed to cognitive performance in women with chronic pain-related TMD. Although the other variables also contributed to the pain experience, they were likely to influence aspects other than cognition.

Interestingly, in the comparison between the conditions of pain-related TMD, there was no difference between myalgia and arthralgia regarding the variables studied, reinforcing the idea that more important than the diagnostic subtype is the experience of pain, as demonstrated in the regression analysis. This result allowed the verification of predictor variables compared to the independent variable (cognitive performance) to be performed in a single group for the sample studied. Thus, the results may be extrapolated to patients with painful conditions related to TMD, i.e., worse cognitive performance is expected in patients with higher pain intensity and presence of pain catastrophizing.

Pain catastrophizing may be defined as an exaggerated negative mental perception of the presence of pain and the possibility of reliving this experience in the future. It is characterized by a tendency to magnify the value of pain, experience thoughts focusing on the inability to inhibit it, and feelings of helplessness in the context of pain, and is typically associated with dysfunctional processes of care, evaluation, coping, and hyperactivation of neural areas responsible for the intensity and chronicity of pain.^6 , 16 , 23 , 24 , 25^ Its presence can become a mental habit and lead to worsening of the TMD condition by intensifying the fear-avoidance model of chronic pain.^26^ Moreover, intensification in the frequency of this type of thinking can lead to fear of pain, with behavioral manifestations such as avoidance of certain activities in order to preserve the area of pain. However, these behaviors commonly result in disability or functional disability due to diminished musculoskeletal use, which, in turn, further contribute to the chronicity of pain.^6^ Schütze, et al.^27^ found that catastrophizing accounted for 7% to 41% of variations in pain severity as such thoughts are typically related to areas of the brain associated with pain processing, attention to pain, motor activity, and aspects of emotion and cognition.^6 , 23^ Strategies aimed at raising awareness and reducing negative mental habits related to pain should be considered when identifying the presence of catastrophic thoughts in patients with chronic pain-related TMD.

A systematic review by Yin, et al.^28^ (2020) showed that patients with pain-related TMD exhibited changes in the brain pathways responsible for the perception and interpretation of pain, including the trigeminal, thalamus-cortex somatosensory system, and the lateral and medial pain systems, which play a role in the processing of cognitive information. Moreover, a series of dysfunctional adaptations in areas involving the periaqueductal gray matter and the descending inhibition system of pain were also observed.^29^ TMD patients also exhibited altered brain activations in response to innocuous and painful stimuli when compared with healthy controls, reinforcing the idea of changes in central processing and the occurrence of central sensitization.^28 - 30^

A recent study showed the presence of orofacial pain and associated potential causes in patients diagnosed with dementia and/or cognitive impairment.^31^ Chronic pain may lead to activation of certain brain regions that participate in cognitive processes related to attention, memory, and learning, resulting in cognitive impairment, as we showed in this study.^22^

The central neural pathways commonly involved in nociceptive and cognitive processing (somatosensory cortex region, limbic system components etc.) are associated, especially in chronic pain, although the exact mechanisms involved in this complex relationship are still unclear.^1 , 7 , 8 , 13^ Recent studies showed that the presence of chronic pain contributes to the acceleration of memory decline and increases the chances of dementia, in addition to being a risk factor for premature death.^32 - 34^

Some confounding variables such as the continuous use of medications (antidepressants, anticonvulsants, and muscle relaxants), presence of comorbidities, and specific symptoms related to anxiety and depression, may also influence outcomes. Thus, mental health disorders, which are frequently observed in patients with pain-related TMD and chronic pain, also play a role in impaired cognitive performance, caused by medications use or by the chronic pain condition.^13 , 32^ However, some studies have also shown that patients with chronic severe pain exhibit impaired basic neurocognitive functions, regardless of the presence of depressive symptoms and medication use.^35^

The relationship between chronic pain and cognitive performance can affect patient’s daily activities, including those related to attention, memory, and executive function, thus significantly affecting their social relationships as well as other dimensions of life (labor, family, etc.).^8 , 13 , 22^ Therapeutic strategies involved with pain education depend on the patient’s understanding and, in the case of impaired cognitive performance, may result in low response rates due to a lack of complete understanding of the measures that should have been adopted. Since pain education is a primary step in the treatment of pain-related TMD, alternative approaches that reinforce such orientations and ensure full understanding and execution are essential in patients exhibiting impaired cognitive performance. Perhaps, individuals who have not adhered to treatment, especially self-management strategies, have not fully understood the condition in which they fit or how they should proceed with home care.

Therefore, the findings of this study, as well as with previous evidence,^22 , 23^ highlight the importance of evaluating cognitive impairment in patients with chronic pain-related TMD, based on the understanding that the neural pathways common to these two experiences are focused on pain processing and, therefore, may also impair cognition. Pain catastrophizing and higher pain intensity levels can predict the appearance of cognitive deficits in patients with chronic pain-related TMD and, upon identification of this triad (catastrophizing, high pain level, and impaired cognitive performance), professionals should design strategies that first aim to reduce catastrophic thoughts and ensure complete understanding of the condition of chronic pain and, later, focus on changing behaviors and identifying worsening, predisposing, and/or perpetuating factors to promote pain education.^13 , 22 , 23 , 27 , 30^

### Study limitations

This was a cross-sectional study that aimed to track relationships between painful and psychological TMD variables and cognitive performance. It was conducted in the dental clinic during the undergraduate students’ academic year; thus, the possibilities of rigorous control for a robust study were limited. Future studies should present larger samples, include a control group, observing and controlling other present body pains, as well as considering the duration of pain as a predictor of the analyzed variables to obtain results that can be scientifically extrapolated.

## Conclusion

The results show that the high intensity of pain and the presence of catastrophizing can predict impaired cognitive performance in women with chronic pain-related TMD, and with the mental dimensions of memory, attention, and executive/visuospatial function being compromised; therefore, proving to be an important topic to more robust scientific investigations. Cognitive deficit can influence the patient’s response to pain education strategies, which depend on the understanding of the condition and actions aimed at behavioral changes. Therefore, patients’ refractory to this type of approach may present a cognitive performance lower than expected because of chronic pain experience, and do not respond to pain education, since aspects such as memory and attention are compromised. Therefore, alternative pain education strategies should be identified, delivered objectively, and reinforced frequently in patients with chronic pain-related TMD exhibiting catastrophizing and high pain intensity. Strategies aimed at psychosocial dimensions such as changes in exaggerated negative mental habits should also be included in this process.

## References

[1] Domenichiello AF, Ramsden CE (2019). The silent epidemic of chronic pain in older adults. Prog Neuropsychopharmacol Biol Psychiatry.

[2] Dahlhamer J, Lucas J, Zelaya C, Nahin R, Mackey S, DeBar L (2018). Prevalence of chronic pain and high-impact chronic pain among adults - United States, 2016. MMWR Morb Mortal Wkly Rep.

[3] International Classification of Orofacial Pain, 1st edition (ICOP) (2020). Cephalalgia.

[4] Vasconcelos FH, Araújo GC (2018). Prevalence of chronic pain in Brazil: a descriptive study. Br J Pain.

[5] Slade GD, Ohrbach R, Greenspan JD, Fillingim RB, Bair E, Sanders AE (2016). Painful temporomandibular disorder: decade of discovery from OPPERA studies. J Dent Res.

[6] Bair E, Gaynor S, Slade GD, Ohrbach R, Fillingim RB, Greenspan JD (2016). Identification of clusters of individuals relevant to temporomandibular disorders and other chronic pain conditions: the OPPERA study. Pain.

[7] Ferreira KS, Oliver GZ, Thomaz DC, Teixeira CT, Foss MP (2016). Cognitive deficits in chronic pain patients, in a brief screening test, are independent of comorbidities and medication use. Arq Neuropsiquiatr.

[8] Schiltenwolf M, Akbar M, Hug A, Pfüller U, Gantz S, Neubauer E (2014). Evidence of specific cognitive deficits in patients with chronic low back pain under long-term substitution treatment of opioids. Pain Physician.

[9] Glass JM, Park DC, Minear M, Crofford LJ (2005). Memory beliefs and function in fibromyalgia patients. J Psychosom Res.

[10] Apkarian V, Hashmi JA, Baliki MN (2011). Pain and the brain: specificity and plasticity of the brain in clinical chronic pain. Pain.

[11] Legrain V, Damme SV, Eccleston C, Davis KD, Seminowicz DA, Crombez G (2009). A neurocognitive model of attention to pain: behavioral and neuroimaging evidence. Pain.

[12] Rohling ML, Green P, Allen LM, Iverson GL (2002). Depressive symptoms and neurocognitive test scores in patients passing symptom validity tests. Arch Clin Neuropsychol.

[13] Berryman C, Stanton TR, Bowering KJ, Tabor A, McFarlane A, Moseley GL (2014). Do people with chronic pain have impaired executive function? A meta-analytical review. Clin Psychol Rev.

[14] Suhr JA (2003). Neuropsychological impairment in fibromyalgia: relation to depression, fatigue and pain. J Psychosom Res.

[15] Sehn F, Chachamovich E, Vidor LP, Dall-Agnol L, Souza IC, Torres IL (2012). Cross-cultural adaptation and validation of the Brazilian Portuguese version of the pain catastrophizing scale. Pain Med.

[16] Sampaio Bonafé FS, Marôco J, Duarte Bonini Campos JA (2017). Cross-Cultural Validation of the brazilian portuguese version of the pain vigilance and awareness questionnaire. J Oral Facial Pain Headache.

[17] Schiffman E, Ohrbach R, Truelove E, Look J, Anderson G, Goulet JP, International RDC/TMD Consortium Network, International association for Dental Research, Orofacial Pain Special Interest Group, International Association for the Study of Pain (2014). Diagnostic Criteria for Temporomandibular Disorders (DC/TMD) for clinical and research applications: recommendations of the International RDC/TMD Consortium Network and Orofacial Pain Special Interest Group. J Oral Facial Pain Headache.

[18] Peck CC, Goulet JP, Lobbezoo F, Schiffman EL, Alstergren P, Anderson GC (2014). Expanding the taxonomy of the diagnostic criteria for temporomandibular disorders. J Oral Rehabil.

[19] Ohrbach R, Pereira FJ, Gonçalves DA (2016). Diagnostic criteria for temporomandibular disorders: assessment instruments (Brazilian Portuguese). Version 15 May 2016.

[20] Freitas S, Simões MR, Martins C, Vilar M, Santana I (2010). Adaptation studies of the montreal cognitive assessment (MoCA) to the Portuguese population. Aval Psicol.

[21] Caumo W, Antunes LC, Elkfury JL, Herbstrith EG, Busanello Sipmann R, Souza A (2017). The Central Sensitization Inventory validated and adapted for a Brazilian population: psychometric properties and its relationship with brain-derived neurotrophic factor. J Pain Res.

[22] Baker KS, Georgiou-Karistianis N, Gibson SJ, Giummarra MJ (2017). Optimizing cognitive function in persons with chronic pain. Clin J Pain.

[23] Baker KS, Gibson S, Georgiou-Karistianis N, Roth RM, Giummarra MJ (2016). Everyday executive functioning in chronic pain: specific deficits in working memory and emotion control, predicted by mood, medications, and pain interference. Clin J Pain.

[24] Ossipov MH, Dussor GO, Porreca F (2010). Central modulation of pain. J Clin Invest.

[25] Zeidan F, Baumgartner JN, Coghill RC (2019). The neural mechanisms of mindfulness-based pain relief: a functional magnetic resonance imaging-based review and primer. Pain Rep.

[26] Vlaeyen JW, Linton SJ (2000). Fear-avoidance and its consequences in chronic musculoskeletal pain: a state of the art. Pain.

[27] Schütze R, Rees C, Preece M, Schütze M (2010). Low mindfulness predicts pain catastrophizing in a fear-avoidance model of chronic pain. Pain.

[28] Yin Y, He S, Xu J, You W, Li Q, Long J (2020). The neuro-pathophysiology of temporomandibular disorders-related pain: a systematic review of structural and functional MRI studies. J Headache Pain.

[29] Roy A, Wang WE, Ho RL, Ribeiro-Dasilva MC, Fillingim RB, Coombes SA (2018). Functional brain activity during motor control and pain processing in chronic jaw pain. Pain.

[30] Weissman-Fogel I, Moayedi M, Tenenbaum HC, Goldberg MB, Freeman BV, Davis KD (2011). Abnormal cortical activity in patients with temporomandibular disorder evoked by cognitive and emotional tasks. Pain.

[31] Delwel S, Scherder EJ, Baat C, Binnekade TT, van der Wouden JC, Hertogh CMPM (2019). Orofacial pain and its potential oral causes in older people with mild cognitive impairment or dementia. J Oral Rehabil.

[32] Whitlock EL, Diaz-Ramirez LG, Glymour MM, Boscardin WJ, Covinsky KE, Smith AK (2017). Association between persistent pain and memory decline and dementia in a longitudinal cohort of elders. JAMA Intern Med.

[33] Cole LJ, Farrell MJ, Duff EP, Barber JB, Egan GF, Gibson SJ (2006). Pain sensitivity and fMRI pain-related brain activity in Alzheimer's disease. Brain.

[34] Macfarlane GJ, Barnish MS, Jones GT (2017). Persons with chronic widespread pain experience excess mortality: longitudinal results from UK Biobank and meta-analysis. Ann Rheum Dis.

[35] Wolrich J, Poots AJ, Kuehler BM, Rice AS, Rahman A, Bantel C (2014). Is number sense impaired in chronic pain patients?. Br J Anaesth.

